# Pneumocoele sinus: Computed tomographic findings and surgical approach in a 2‐year‐old Cob filly

**DOI:** 10.1002/evj.70226

**Published:** 2026-07-05

**Authors:** Sarah L. Thompson, Manon W. J. Peeters, Gabriel Manso‐Díaz, Andrew R. Fiske‐Jackson

**Affiliations:** ^1^ Equine Diagnostic Imaging The Royal Veterinary College Hatfield UK

**Keywords:** advanced imaging, airway, endoscopy, horse, stertor, transendoscopic laser

## Abstract

**Background:**

Pneumosinus dilatans and pneumocoele sinus describe abnormal hyperpneumatisation of paranasal sinuses, with the latter being accompanied by thinning of bony walls. Although recognised in human and equine medicine, to the authors' knowledge, this condition's computed tomography (CT) findings and successful surgical approach in horses have not previously been reported in conjunction. Advanced imaging, particularly CT, is pivotal for identifying abnormal sinus expansion and associated nasal obstruction.

**Study Design:**

Case report.

**Clinical Summary:**

A 2‐year‐5‐month‐old Cob filly presented with a 1‐month history of progressive inspiratory stertor and weight loss, unresponsive to systemic corticosteroids. Physical examination revealed marked inspiratory stertor. Endoscopy suggested narrowing of nasal passages but was subjective and did not identify the underlying cause. Standing CT of the head demonstrated extensive, bilateral dilation of the dorsal and ventral conchal sinuses with marked thinning of axial sinus walls. The nasomaxillary apertures were occluded, and the dorsal and middle nasal meatuses were markedly narrowed or obliterated. No evidence of sinusitis or other intraluminal disease was present. Transnasal diode‐laser fenestrations into the dorsal and ventral conchal sinuses established permanent communication with the nasal cavity. Foley catheters were placed for postoperative flushing. A temporary tracheostomy ensured airway patency. Seventeen days postoperatively, the filly showed complete resolution of inspiratory stertor. Endoscopic evaluation revealed large, epithelialised fistulae and restoration of normal nasal meatus dimensions. Long‐term follow‐up confirmed persistent clinical normality with no recurrence.

**Main Limitations:**

Single case report.

**Conclusions:**

CT imaging was essential for diagnosis and surgical planning for a case of pneumocoele sinus in a horse. Creation of large, permanent fenestrations between the conchal sinuses and nasal cavity successfully equalised sinus pressure, reversed nasal obstruction, and resulted in an excellent long‐term outcome. This condition should be considered as a differential diagnosis in equine upper airway obstruction.

## 1 INTRODUCTION

Excessive paranasal sinus pneumatisation is referred to as pneumosinus dilatans or pneumocoele sinus in the human literature.^1^ These terms are used to describe expansion of one or more paranasal sinuses with air (hyperpneumatisation) with or without bone or mucous membrane changes to the walls of the sinus compartment.^1^ When thinning (focal or generalised) of the bony wall of a sinus is present, the condition is referred to as a pneumocele sinus and when there is no change to the bony walls the condition is referred to as pneumosinus dilatans.^2^, ^3^ In human medicine pneumosinus dilatans is described with or without local compressive signs^3^, ^4^; therefore, it can be either symptomatic or asymptomatic.^2^ In human medicine pneumosinus dilatans and pneumocele sinus are both referred to as an air cyst^2^ a well described, but uncommon condition. Previous reports of a sinus pneumocele in horses include a 2005 case report by Breuer et al. detailing radiographic and histopathologic findings, and a study by Charles et al. describing CT findings and clinical outcomes in eight affected horses.^5^, ^6^

In human medicine, advanced imaging is essential for the diagnosis of excessive paranasal sinus pneumatisation, as patients can have a normal nasal endoscopic examination.^4^ In equine medicine, computed tomography (CT) allows for specific 3D imaging of the intricate equine paranasal sinuses and can aid surgical planning.^7^ Multiplanar reconstructions of CT images demonstrate the spatial relationships between paranasal sinuses and neighbouring structures in the cavities of the head.

This case study describes the clinical presentation, imaging findings, surgical approach and successful outcome of an unusual case of excessive paranasal sinus pneumatisation/pneumosinus dilatans in a 2‐year‐old Cob filly.

## 2 PRESENTING SIGNS

A 2‐year 5‐month‐old Cob filly initially presented to the referring practice 1 month prior to referral for marked inspiratory stertor and weight loss. The filly was treated with systemic steroids—an initial 0.03 mg/kg dose of intravenous dexamethasone followed by daily prednisolone (1 mg/kg, per os [PO], SID) for 1 week with no improvement seen. The endoscopic examination performed by the referring veterinarian was indicative of suspected narrowing of the nasal passage and pharynx region. On admission to the Royal Veterinary College (RVC), the filly weighed 210 kg, had a normal physical examination apart from a stertorous snoring noise during inspiration.

## 3 IMAGING FINDINGS

The filly was referred to the RVC Equine Referral Hospital for a CT examination of the head, which was performed standing, under sedation, using a standardised protocol (incremental doses of detomidine 0.01 mg/kg IV). The axial CT was performed with a 16‐slice multi‐detector CT scanner (GE Healthcare) using 120 kV and 400 mAs with a 1.25 mm slice thickness and an inter‐slice interval of 1.25 mm. The images were reconstructed using bone and soft tissue algorithms.

The CT study demonstrated bilateral marked dilation of the dorsal conchal and ventral conchal sinuses; the dorsal conchal sinus was larger than the ventral conchal sinus with resultant occlusion of the nasomaxillary aperture, dorsal and middle nasal meatuses (Figure 1). The axial walls of both the dorsal and ventral conchal sinuses were severely thinned. These findings were bilateral and symmetrical. However, the remaining bony wall margins of the sinuses appeared normal. The ventral walls of these sinuses were displaced ventrally coming into contact with the apices of the maxillary 09 teeth, and the rostral walls were expanded rostrally, causing a reduction in the volume of the dorsal nasal conchal bullae. The caudal extent of the dorsal and ventral nasal conchal bullae were located at the level of the maxillary 08 teeth.

**FIGURE 1:**
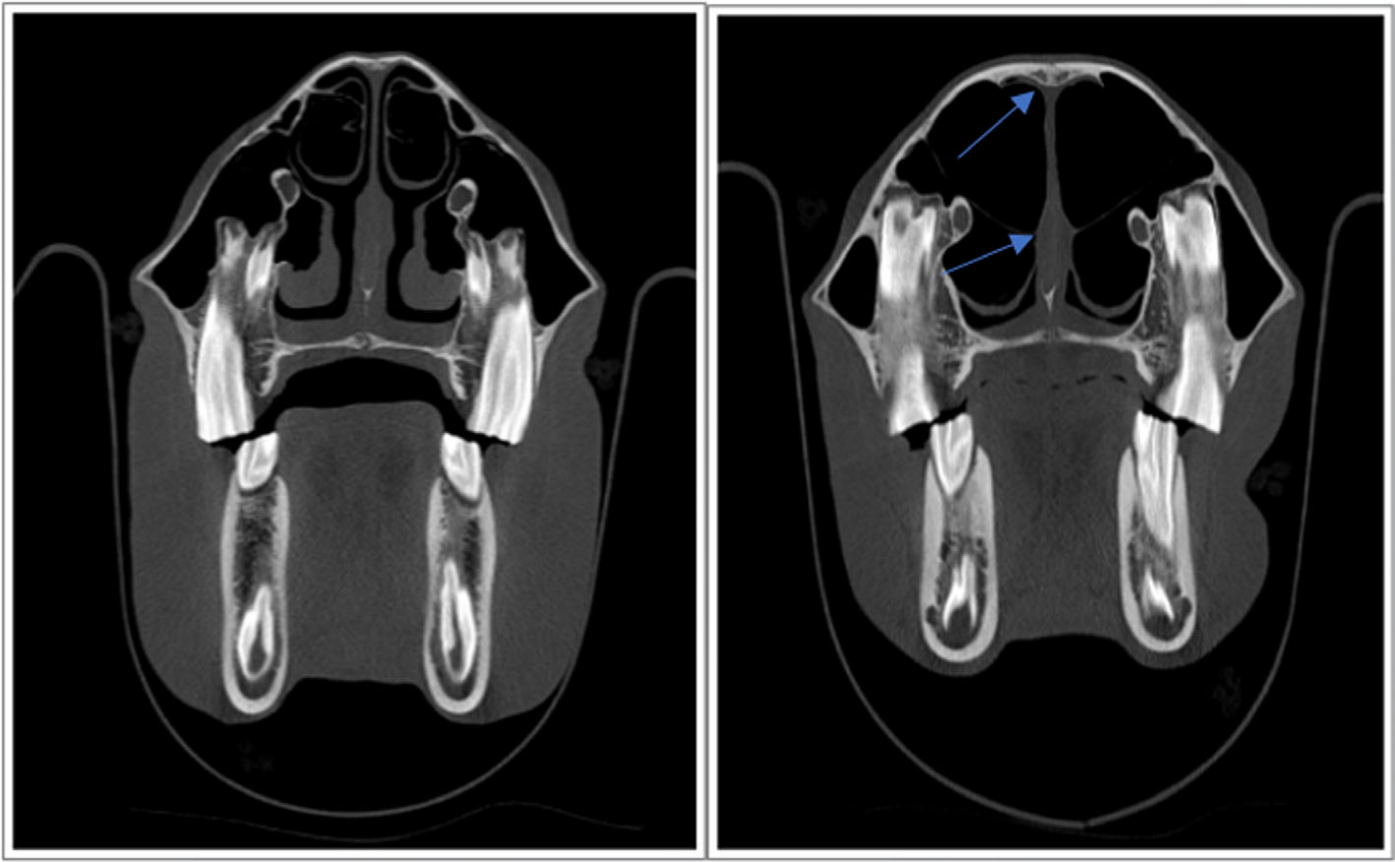
The image on the left is a control at the level of the 09 maxillary teeth and the image on the right is the case. There is marked dilation of the dorsal conchal and ventral conchal sinuses. The arrows indicate the secondary marked narrowing of the dorsal and middle meatuses.

The dorsal and ventral conchal sinuses were also enlarged axially, thereby markedly reducing the cross‐sectional area of the middle and dorsal nasal meatuses from the level of the maxillary 08 teeth to the level of the 11 teeth. The aberrant nasal meatuses were compressed and irregular in shape. A small volume of gas was present in the right dorsal nasal meatus and the middle meatuses at the level of the maxillary 08 teeth. However, at the level of the adjacent 09s there was complete loss of the normal gas attenuation within the dorsal nasal meatuses and the left middle nasal meatus. The nasomaxillary apertures were not visible on CT. The ventral meatuses remained patent.

## 4 ENDOSCOPIC FINDINGS

The filly underwent an upper airway endoscopy after her CT examination which confirmed enlargement of the dorsal and ventral conchae with narrowing of the middle and obliteration of the dorsal meatuses. The ventral meatuses were patent and there appeared to be adequate space for airflow. During normal breathing the walls of the ventral conchae appeared mobile thereby obliterating the middle meatus during inspiration because of the negative pressure, returning to their original position during expiration (Figure 2).

**FIGURE 2:**
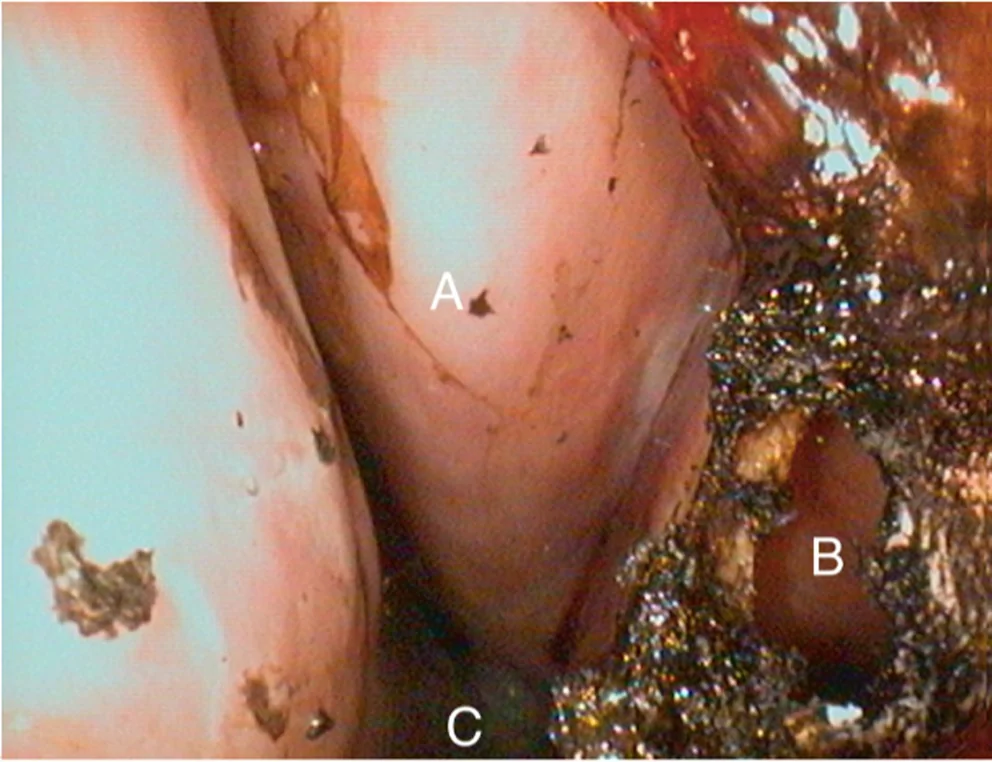
Intra‐operative view in the left nasal passage, for orientation the nasal septum is to the left of the image. The ventral wall of the dorsal conchal sinus is still intact (A). Endoscopic laser fenestration of the ventral conchal sinus (B) has been performed with minimal haemorrhage. (C) Indicates the entrance to the nasopharynx.

## 5 SURGICAL APPROACH

The decision was made to fenestrate into both the dorsal and ventral conchal sinuses via the nasal cavity bilaterally to equalise the pressure between the nasal cavity and sinuses. The horse was sedated using a standard sedation protocol of detomidine (0.01 mg/kg intravenously [IV]), butorphanol (0.02 mg/kg IV), and acepromazine (0.02 mg/kg IV) before being positioned in stocks, and throughout the procedure was given incremental doses of detomidine and butorphanol at 0.005 mg/kg and 0.01 mg/kg, respectively. The filly received perioperative flunixin meglumine (1.1 mg/kg IV, BID) and oxytetracycline (8 mg/kg IV, BID), which were continued during hospitalisation. Prior to the surgical procedure, a temporary tracheostomy was performed to ensure adequate airflow during and after the procedure. The first attempt to fenestrate into the dorsal and ventral conchal sinuses was performed via a transnasal approach using an endoscopic diode laser. A splash block using 20 mL lidocaine (2%) at each site resulted in sufficient analgesia to perform the procedure. To control haemorrhage, phenylephrine was sprayed topically over the fenestration site. Using the measurements from the CT scan, the diode laser was used to fenestrate into the dorsal and ventral conchal sinuses 25 cm from the nares. Approximately 2 cm diameter fenestrations were made into the ventral aspect of the dorsal conchal sinuses and the dorsal aspect of the ventral conchal sinuses. Haemorrhage occurred during fenestration of the right dorsal conchal sinus, which compromised visibility for 10–15 min, requiring a corresponding pause during the surgery. No significant haemorrhage occurred during fenestration of the other sites. Repeat endoscopic evaluation the day after the procedure revealed the fenestrations were of insufficient size deemed required to ensure long term patency.

Bilateral sinoscopy was performed the following day to provide improved access to enlarge the fenestrations. Local analgesia at the trephine sites was provided using subcutaneous infiltration of 10 mL 2% mepivacaine hydrochloride. Right and left conchofrontal trephine portals were made, using a 25 mm Galt trephine. The fenestrations into the dorsal conchal sinus were visualised and enlarged using the endoscopic‐guided diode laser to an approximate diameter of 4 cm. The maxillary septal bullae were fenestrated using a Noyes forceps and then enlarged using angled Ferris Smith rongeurs. This allowed passage of the endoscope into the ventral conchal sinus and visualisation of the fenestrations into these sinuses from the nasal cavity. In a similar fashion these were enlarged to approximately 4 cm diameter. Foley catheters were placed into both conchofrontal sinuses and secured using a roman sandal suture pattern using USP 0 nylon. The skin was closed using USP 0 nylon in a simple interrupted suture pattern.

The sinuses were flushed using 3 L 0.9% sodium chloride solution three times the day after surgery to ensure patency of the fenestrations. Repeat endoscopy of the nasal passages 2 days following the sinoscopy demonstrated adequately sized fenestrations in both right and left dorsal and ventral conchal sinuses. The nasal passages appeared more patent on transnasal endoscopy due to less movement associated with breathing. Both Foley catheters were removed and the sinoscopy incisions were closed using USP 0 nylon in a simple interrupted suture pattern. The tracheostomy tube was removed; a reduction in inspiratory stertor and even airflow from both nostrils was noted. The filly was discharged on phenylbutazone (2.2 mg/kg, PO, BID) and doxycycline (10 mg/kg, PO, BID) both for 5 days.

## 6 OUTCOME

Seventeen days post discharge, the horse was readmitted for repeat endoscopic evaluation and suture removal. The owner reported complete cessation of the inspiratory stertor, which was confirmed on examination. Upper airway endoscopy showed large, epithelialised fistulae between the nasal cavity and dorsal and ventral conchal sinuses bilaterally (Figure 3). Subjectively, normal‐sized dorsal, middle, and ventral meatuses were present with no visible movement of the dorsal or ventral conchae walls; normal airflow was evident with no inspiratory stertor. The tracheostomy site was granulating as expected and sealed over. The sutures from both sinoscopy portals were removed and both sites healed by primary intention. Follow‐up 2 years and 4 months postoperatively, the horse was free of any clinical signs.

**FIGURE 3:**
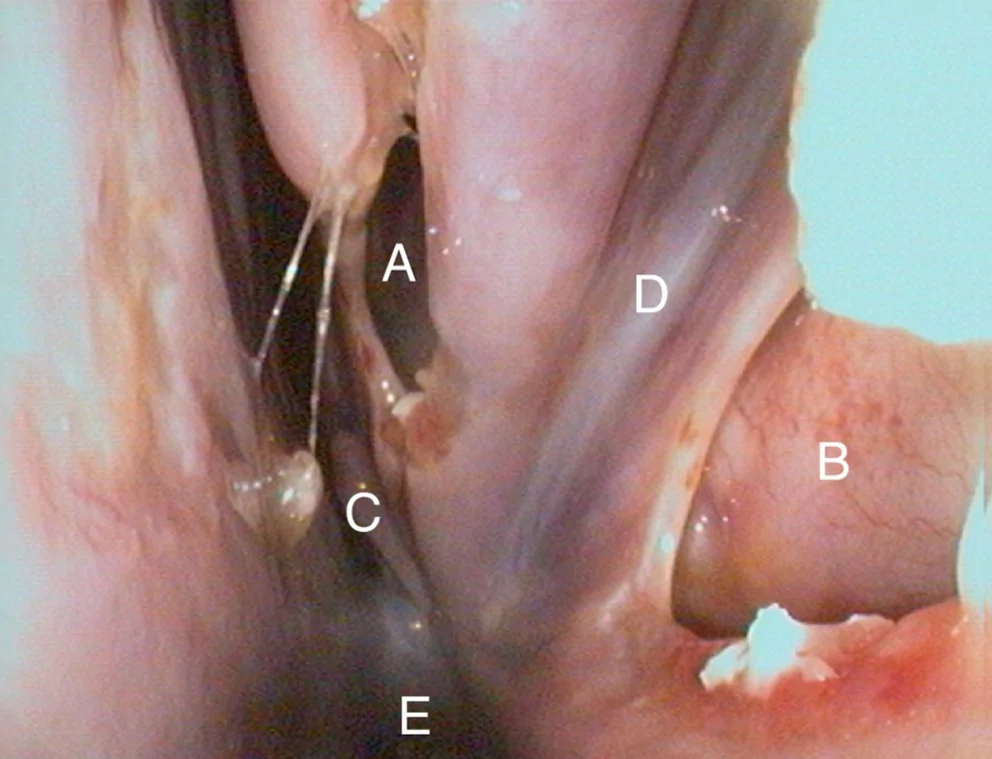
The transnasal endoscopic view in the left nasal passage of the dorsal and ventral sinus fenestrations 17 days post sinoscopy. Fenestration into the dorsal conchal sinus (A). Fenestration into the ventral conchal sinus (B). Rostral aspect of the ethmoid turbinates (C). Nasomaxillary opening (D). Entrance to the nasopharynx (E).

## 7 DISCUSSION

Thin‐slice three‐dimensional CT imaging provides improved visualisation of indirect signs of compressive disorders that are not appreciable with standard radiographic imaging. One of the many benefits of CT is its high spatial resolution; this allows for detailed evaluation of bone structures and gas‐soft tissue interfaces. This benefit pairs CT imaging well with assessing the paranasal sinus and identifying any subtle changes in the nasal structures which may not be appreciated on radiography. CT imaging is suitable for assessing bone lesions in the head and has superior contrast resolution to radiographs when evaluating soft tissue structures.^2^, ^8^ Previous authors have found that the majority (61%) of horses have the caudal limit of the dorsal nasal conchal bulla extending to the maxillary triadan 10s, with only 3% of horses having the caudal limit extend to the maxillary tridan 08s.^7^ Interestingly, in this case, the dorsal nasal conchal bullae extended only to the level of the maxillary triadan 08s. This could be explained by the age of the patient, as younger horses have smaller dorsal and ventral nasal conchal bullae; therefore, extending less far caudally.^7^ It could also be argued that the reduction of the caudal limit of the nasal conchal bulla could be due to the dilation of the dorsal conchal sinus. Knowledge of anatomy and normal sizes of the paranasal sinuses is imperative to recognise and appreciate pathology during CT examination.

Pneumosinus dilatans has been reported in both young and elderly human patients.^1^ The aetiology is undetermined but different theories have been proposed. These include a one‐way valve causing a build‐up of pressure, sinusitis with gas producing bacteria, hormonal imbalance causing bone remodelling, congenital defects and rupture of a mucocele.^2^, ^3^ It has also been questioned whether pneumosinus dilatans and pneumocoeles have separate aetiologies or whether one is a progression of the other.^2^

A further possible cause of narrowing of the nasal meatuses in the equine patient is remodelling of the nasal conchae or swelling of the ventral nasal conchae, commonly seen in horses with sinusitis, sinus cysts or sinus neoplasia.^9^ The deformation of the nasal conchae can be severe and cause occlusion of the ipsilateral nasal cavity and displace the nasal septum towards the contralateral side. These horses often present with an inspiratory stridor and reduction of airflow from the ipsilateral nostril.^9^ In the current case, the CT scan enabled straightforward exclusion of sinusitis as a possible cause of the constricted nasal meatuses.

The equine upper airway is designed to prevent collapse during inhalation. Any failure in the maintenance of stability results in obstruction of the airway with an associated inspiratory stertor or stridor during inspiration.^10^ The nasal cavity is rigidly supported by cartilage and bone. Small changes in diameter occur due to the presence of sympathetic innervated capacitance vessels, which constrict during exercise. Excessive dilation of these vessels can obstruct the nasal cavity completely when there is injury to the sympathetic trunk, as in cases of Horner's syndrome.^11^ Intranasal injection with phenylephrine can reduce nasal mucous membrane thickness by causing vasoconstriction.^12^ Phenylephrine was used intraoperatively in our case to arrest bleeding by causing vasoconstriction.

Haemorrhage is a well‐known complication encountered during fenestration between the nasal cavity and the sinus cavities.^13^ During the first procedure an attempt was made to fenestrate through the walls of the dorsal and ventral conchal sinuses via a transnasal endoscopically guided diode laser technique; this was partially successful. The disadvantage of this technique was the marked reduction in visualisation once haemorrhage of the fenestration site occurred. Therefore, the decision was made to perform bilateral conchofrontal sinoscopy to visualise and enlarge the fistulae via the sinuses. This allowed significantly improved visualisation and allowed placement of a Foley catheter to allow subsequent flushing of the sinuses to maintain patency and avoid occlusion with blood clots. Performing the procedure via the sinuses also allowed enlargement of the existing fenestrations unimpeded by mild haemorrhage. Larger fenestrations are recommended to reduce the risk of adhesion formation between the transected edges of the fistulae.^14^ Maintenance of the fistulae to become permanent was deemed essential to prevent recurrence of the clinical signs.

In this case the inspiratory stertor was apparent even at rest. The frequency and intensity of the stertor had increased during the week prior to presentation. We postulate the sinuses dilated to the point where they obstructed the sinonasal ostia and therefore could not deflate. The resultant reduction of the nasal cavity caused marked inspiratory stertor. The authors theorise that this ‘one‐way valve’ effect may cause intermittent obstruction, permitting mucus drainage from the sinus but failing to remain open long enough to prevent the accumulation of air. The aim of the surgery was to create suitably large fenestrations of the left and right dorsal and ventral conchae which would bypass the one‐way‐valve which had developed thereby allowing deflation of the sinuses. In humans, similar surgical procedures have been carried out. Both antrostomies and permeabilisation of the sinus openings ensure drainage of blocked or aerated sinuses; both equilibrate the intrasinus pressure with that of the nasal cavity.^2^, ^3^

Various techniques in the equine patient have been described for obtaining a patent sinonasal ostium, including balloon sinuplasty and transendoscopic diode laser fenestration.^13^, ^15^ Balloon sinuplasty only provides effective dilation of the caudal sinonasal canal and will not prevent ballooning of the ventral and dorsal concha. It would also be less likely to provide an adequate size of sinonasal opening. Another procedure that was considered which may have created adequate transnasal fenestration was a transnasal conchotomy of the ventral conchal sinus, a technique described by Bach et al.^14^ However, the lack of rigidity of the tissue would have made this technique more challenging.

To the authors' knowledge, this is the first published case report of the CT findings of pneumocoele sinus in conjunction with the successful surgical approach in the horse. Knowledge of the anatomy of the paranasal sinuses and the normal variants was important requirements for the understanding of pathological processes; CT allows far greater scrutiny of images in such cases. The CT findings were consistent with the clinical features. The major CT findings of this case were bilateral severe dilation of the dorsal and ventral conchal sinuses with subsequent occlusion of the nasomaxillary opening and narrowing of the nasal meatuses. The knowledge of pneumocoele sinus and its possible presentation allows us to highlight this abnormality as a differential in upper airway disorders. This case also demonstrated that fenestration of the walls of the dilated sinuses into the nasal cavity allowed equalisation of pressure thereby reversing their obstruction of the nasal meatuses and consequent resolution of the clinical signs.

## 8 CONCLUSION

This case report is the first to comprehensively describe the clinical presentation, endoscopic and CT findings as well as the surgical approach to successfully treating pneumocoele sinus in a horse with 2 years and 4 months follow‐up.

## FUNDING INFORMATION

Not applicable.

## CONFLICT OF INTEREST STATEMENT

The authors declare no conflicts of interest.

## AUTHOR CONTRIBUTIONS

**Sarah L. Thompson:** Conceptualization; investigation; writing – original draft; writing – review and editing; visualization; data curation. **Manon W. J. Peeters:** Writing – review and editing; investigation; writing – original draft; data curation. **Gabriel Manso‐Díaz:** Validation; writing – review and editing; resources. **Andrew R. Fiske‐Jackson:** Validation; writing – review and editing; investigation; formal analysis; supervision; visualization; methodology; resources.

## DATA INTEGRITY STATEMENT

Sarah L. Thompson had full access to all the data in the study and takes responsibility for the integrity of the data and the accuracy of the data analysis.

## ETHICAL ANIMAL RESEARCH

Research ethics committee oversight not required by this journal: retrospective study of clinical records.

## INFORMED CONSENT

Signed consent was received from the owner of the pony for the use of the records for research.

## Data Availability

The data that support the findings of this study are available upon reasonable request from the corresponding author. Open data sharing exemption granted by the editor.
